# Omentectomy Prevents Metabolic Syndrome By Reducing Appetite and Body Weight In A Diet-Induced Obesity Rat Model

**DOI:** 10.1038/s41598-018-19973-z

**Published:** 2018-01-24

**Authors:** Inmaculada García-Ruiz, Pablo Solís-Muñoz, Daniel Fernández-Moreira, Montserrat Grau, Maria Teresa Muñoz-Yagüe, José A. Solís-Herruzo

**Affiliations:** 1 Gastroenterology and Hepatology Laboratory, Research Institute, University Hospital “12 de Octubre”. Universidad Complutense, 28041 Madrid, Spain; 2 Translational Oncology, Instituto de Investigación Hospital 12 de Octubre (i+12), Avda de Córdoba s/n, 28041 Madrid, Spain; 3 Institute of Liver Studies, King’s College Hospital, SE5 9RS London, United Kingdom; 4 Department of Bromatology and Food Hygiene, Military Center of Veterinary of Defense, 28024 Madrid, Spain

## Abstract

Visceral fat deposition is associated with impairment of glucose and lipid metabolism while leptin levels are frequently related to subcutaneous fat area. At present, there is considerable controversy regarding the role of visceral adipose tissue accumulation in the development of metabolic syndrome (MS). Here we show the effects of omentectomy on the liver and MS in a diet induced obesity rat model. Our results reveal that undergoing omentectomy previously the establishment of the diet-induced-obesity reduced significantly body weight gain and avoid the development of MS, including non-alcoholic fatty liver disease. Intriguingly, the significantly lower body weight gain was due to decreased food intake. Omentum drives obesity progression through leptin resistance mediated by C-reactive protein, Interleucin (IL)-6 and high lipolysis activity. Omentum removal reversed immediately the increased plasma levels of CRP and IL-6 and gradually food intake, weight gain, and features of MS in diet-induced-obesity. Omentectomy caused no changes in normal-weigh-rats. This report displays causal mechanism by which omentum promotes obesity and propose omentectomy as a promising procedure in MS prevention.

## Introduction

Epidemiological and physiological studies have demonstrated a strong association between excess of abdominal adipose tissue, both mesenteric and omental fat, and the presence of metabolic risk factors including insulin resistance, impaired glucose tolerance, type 2 diabetes, dyslipidemia, nonalcoholic steatohepatitis, coronary heart disease, and increased circulating proinflammatory proteins^1^. An extensive literature has demonstrated that visceral and subcutaneous fat (SCF) differ metabolically. For example, visceral adipose tissue (VAT) is less sensitive to insulin, shows increased lipolysis, releases more fatty acids (FFAs), and contains more IL-6^2^ and C-reactive protein (CRP) when compared to subcutaneous adipose tissue^3^. Moreover, VAT drains directly into the liver through the portal circulation and, therefore, hepatocytes are exposed to high levels of FFAs from lipolytically active VAT and to proinflammatory factors, such as TNF-α, IL-6, IFNγ, leptin, and CRP. FFAs cause insulin resistance, increase glucose production, impair the ability of hepatocytes to degrade insulin, provoke hyperinsulinemia and consequently increase triglyceride synthesis, hepatic steatosis and non-alcoholic fatty liver disease (NAFLD)^4^. Moreover, FFAs induce nitroxidative stress and inflammation leading to the progression of fatty liver to nonalcoholic steatohepatitis^5^. Likewise, MS is associated with dysregulation in the synthesis and secretion of adipokines and a decrease in insulin sensitizing and anti-inflamatory adiponectin^6^.

Considering the role played by VAT in the pathogenesis of metabolic syndrome (MS), the removal of this tissue might benefit patients with MS, including NAFLD. In fact, omentectomy decreases glucose and insulin levels^7^, whereas a reduction of SCF by liposuction does not improve MS^8^. Weight loss through diet and exercise, which cause preferential VAT loss, has proven effective in improving metabolic diseases^9^, and SCF transplantation into the abdominal cavity has beneficial effects on MS (improves insulin sensitivity and glucose tolerance, reduces body weight).

While the effects of omental fat removal have rarely been studied in animal models of established obesity, to our knowledge the role of omentum in obesity development is unexplored. Furthermore, contradictory data and conclusions have derived from human studies. Hence, we aim to determine the effects of omentectomy on the liver histology and on MS in diet-induced obesity.

## Results

### Omentectomy reduced weight gain and prevented the development of MS and NAFLD in HFD-fed rats

**Figure 1:**
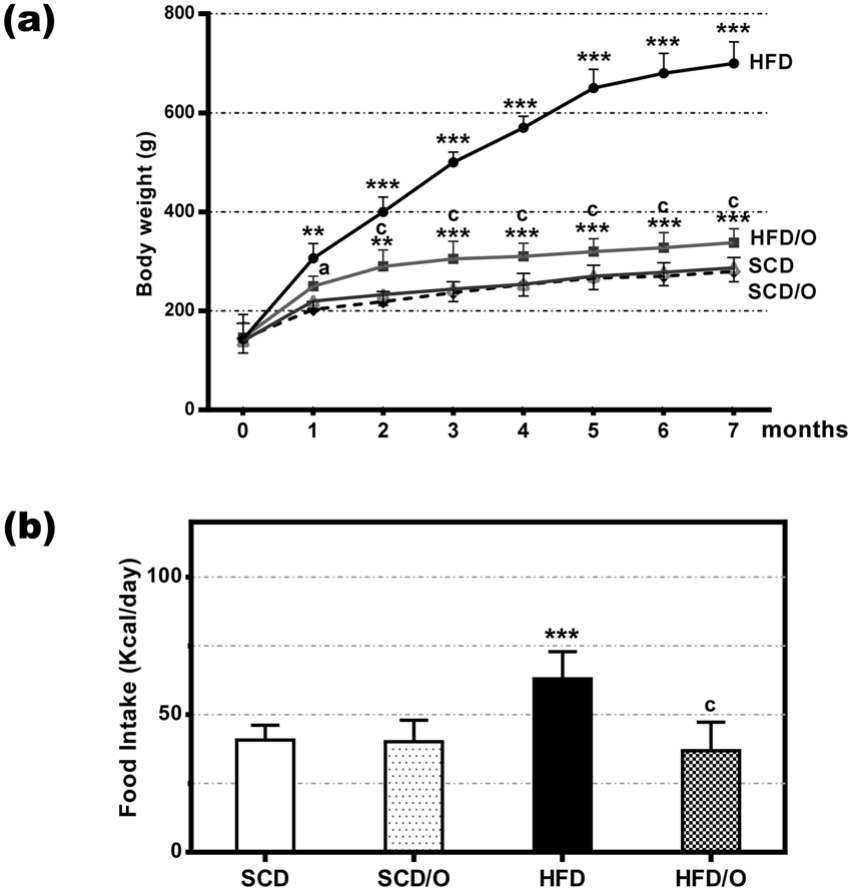
Effect of omentectomy on body weight and food intake, in rats fed a HFD. (**a**) Evolution of body weight in groups 1–4. SCD, rats on a standard chow diet that underwent sham surgery after the acclimatization period (at the age of six weeks); SCD/O, rats on a standard chow diet that underwent omentectomy after the acclimatization period; HFD, rats on a high fat diet. They underwent sham surgery after the acclimatization period; HFD/O, rats on a HFD. These rats underwent omentectomy after the acclimatization period; (**b**) Food intake by the groups 1–4 of rats. This intake was measured at the end of the experimental period. Statistical differences between groups were analyzed by two-way ANOVA. **p < 0.01; ***p < 0.001 effect of diet; (**a**), p < 0.05; (**c**), p < 0.001 effect of surgery.

**Figure 2:**
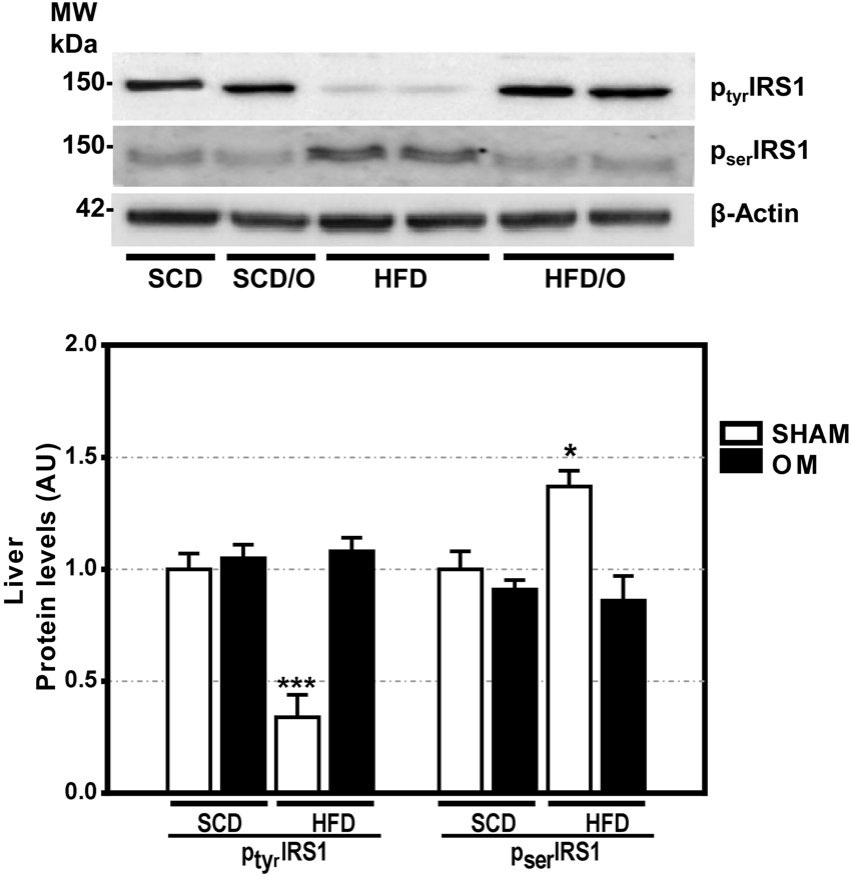
Effect of omentectomy on insulin resistance and leptin resistance, in rats fed a HFD. (**a**) Tyrosine and serine phosphorylation of IRS1 in the liver of rats fed a standard chow diet with omentectomy (SCD/O) and without it (SCD), and in rats on a high fat diet with omentectomy (HFD/O) and without it (HFD). Bar graph illustrating the protein expression of IRS1 is shown at the bottom of the figure. The protein expression in sham group fed a SCD was assumed to be 1. Results are given as mean ± S.D., *p < 0.05; ***p < 0.001 *vs*. SCD.

**Figure 3:**
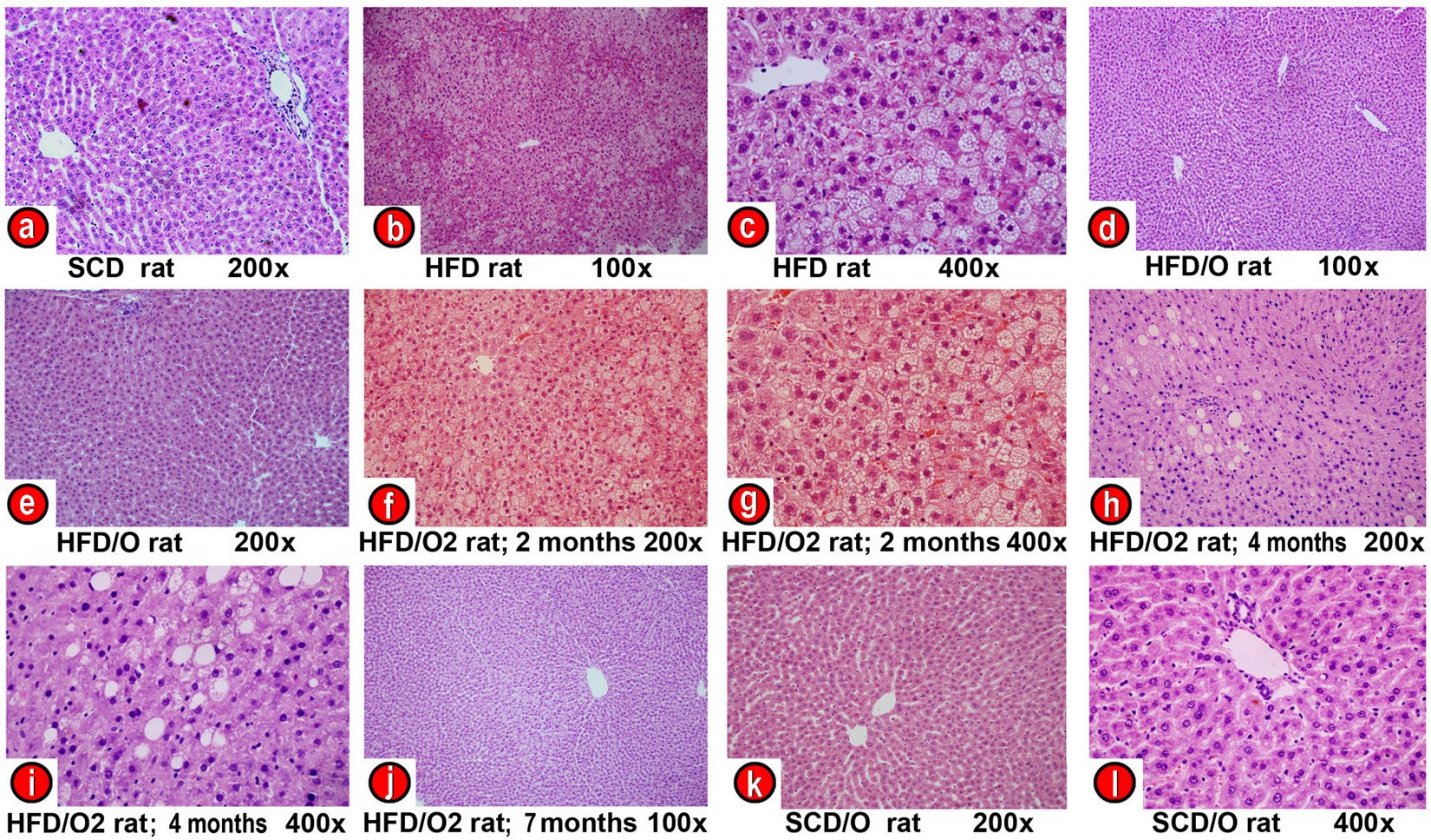
Effects of omentectomy on the liver histology. (**a**) Control rat on a standard chow diet (SCD-rat). (**b,c**) Rat on a high-fat diet for 7 months (HFD-rat). (**d,e**) Rat on a HFD undergoing omentectomy at the age of 6 weeks (HFD/O-rat). (**f,g**) Rat on a HFD for two months (HFD/O2-rat) showing severe hepatic steatosis, mainly microvesicular. After biopsy, omentectomy was carried out in the same rat. (**h,i**) The liver of the same rat on a HFD for four months, two months after omentectomy, showing mild steatosis, mainly macrovacuolar. (**j**) The liver of the same rat on a HFD for 7 months, five months after omentectomy, showing normal liver histology. (**k,l**) Rat on a SCD that underwent omentectomy at the age of 6 weeks. Liver samples were stained with hematoxylin-eosin. Magnification 100x (**b,d,j**), 200x (**a,e,f,h,k**), 400x (**c,g,i,l**).

**Figure 4:**
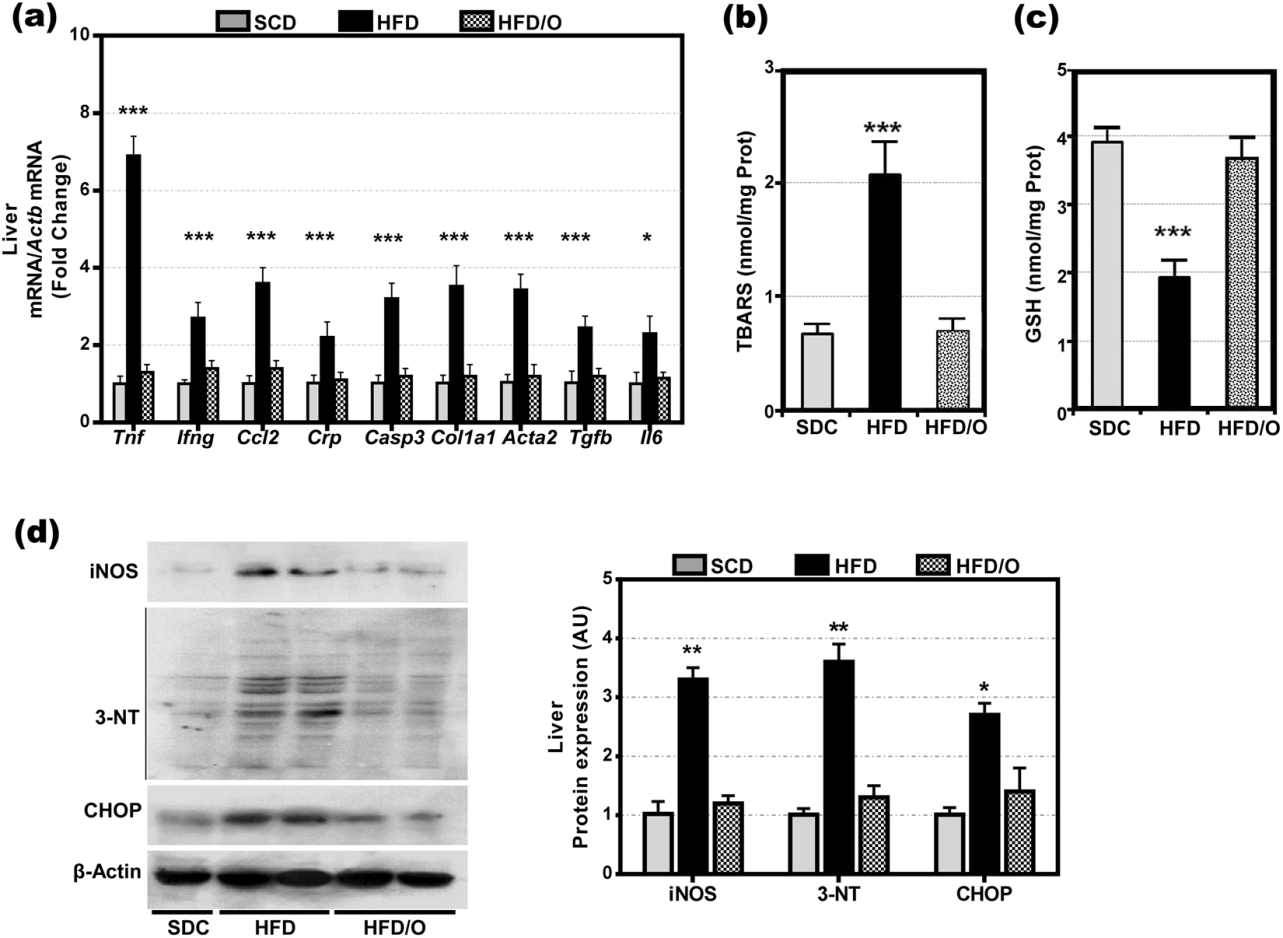
Effects of a HFD and omentectomy on hepatic gene expression of *Tnf, Ifng, Ccl2, Crp, Casp3, Col1a1, Acta2, Tgfb and Il6*, on hepatic TBARS and reduced glutathione levels, and on hepatic protein expression of 3-tyrosine nitrate protein, and CHOP. (**a**) Gene expression of *Tnf, Ifng, Ccl2, Crp, Casp3, Col1a1, Acta2, Tgfb and Il6*. (**b**) Hepatic concentration of TBARS. (**c**) Hepatic concentration of reduced glutathione. (**d**) Protein expression of iNOS, 3-tyrosine nitrated proteins (3-NT), CHOP, and β-actin. Bar graph illustrating the protein expression is shown. Data are mean ± S.D., *p < 0.05; **p < 0.01 ***p < 0.001 *vs*. SCD-rats.

As expected, the HFD caused a marked increase in body weight in HFD-rats as compared with control rats fed a SCD (Fig. 1a). Moreover, HFD-rats developed some features of MS, including elevated plasma glucose, triglyceride, and insulin levels (Table 1). On the other hand, these rats have decreased plasma levels of adiponectin (Table 1). Likewise, hepatic triglycerides were markedly increased in the HFD-rats group indicating the presence of liver steatosis, the hepatic component of MS. Finally, tyrosine phosphorylation of insulin receptor substrate-1 (IRS1) was decreased and serine phosphorylation of the same substrate was increased in the liver of HFD-fed rats (Fig. 2), indicating that these rats developed insulin resistance, a key feature of MS. As compared with SCD-rats (Fig. 3a), the liver of HFD-rats showed a marked accumulation of fat droplets in 50% to 80% of hepatocytes. In 10% to 60% of these hepatocytes, fat was seen mainly as microvesicular droplets (Fig. 3b,c). Although inflammation, ballooning degeneration, and fibrosis were not clearly observed in these rats, the gene expression of *Tnf*, *Ifng*, monocyte chemoattractant protein-1 (*Ccl2*), *Il6* and *Crp* (five inflammatory markers), of *Casp3* (a marker of apoptotic death), and of *Col1a1*, alpha-smooth muscle actin (*Acta2*), and transforming growth factor β1 (T*gfb1*) (three markers of fibrogenesis), was significantly increased in the liver of HFD-rats (Fig. 4a). Moreover, the liver of these obese rats showed evidence of oxidative, nitrosative, and endoplasmic reticulum stress. Thus, HFD-rats had a marked increase in TBARS and a decrease in GSH (Fig. 4b,c). Likewise, protein expression of CHOP (Fig. 4d), a marker of endoplasmic reticulum stress^10^, and of iNOS and 3-tyrosine nitrated proteins (Fig. 4d) was markedly increased in these rats.

**Table 1:** Effects of omentectomy on metabolic syndrome in rats on a high fat diet (HFD).

Parameter	Groups of rats			
	SCD-rats (N, 5)	SCD/O-rats (N, 5)	HFD-rats (N, 5)	HFD/O-rats (N, 5)
Weight gain (g)	130.3 ± 9.1	121.7 ± 8.7	539.6 ± 21.7***	180.2 ± 10.6***^(c)^
Food intake (kcal/day)	40.7 ± 5.4	40.2 ± 7.9	63.0 ± 9.9***	36.9 ± 9.8^(c)^
Plasma glucose (mg/dL)	111 ± 5.8	114 ± 8.4	182 ± 9.4***	121 ± 7.6*^(c)^
Plasma insulin (ng/mL)	2.1 ± 0.4	2.2 ± 0.5	6.3 ± 0.3***	3.2 ± 0.6*^(c)^
Plasma triglycerides (mg/dL)	68.8 ± 6.6	67.1 ± 4.8	187.6 ± 31.0***	57.0 ± 5.1**^(c)^
Plasma adiponectin (µg/mL)	3.2 ± 0.48	3.4 ± 0.6	1.7 ± 0.3***	3.7 ± 0.4 ^(c)^
Perirenal fat Pad (g)	3.1 ± 0.3	2.9 ± 0.3	7.49 ± 0.6***	3.6 ± 0.4***^(c)^
Plasma leptin (ng/mL)	1.5 ± 0.6	1.4 ± 0.8	29.3 ± 2.8***	4.7 ± 0.9***^(c)^
HOMA index	2.3 ± 0.1	2.4 ± 0.2	11.0 ± 0.2***	3.8 ± 0.1***^(c)^
QUICKI	0.27 ± 0.04	0.26 ± 0.06	0.23 ± 0.03***	0.25 ± 0.01^(c)^
FGIR	2.1 ± 0.2	2.2 ± 0.2	1.2 ± 0.1***	1.7 ± 0.2*^(b)^
Hepatic triglycerides (mg/g)	43.0 ± 6.3	42.3 ± 5.3	494.5 ± 6.2***	58.2 ± 5.6**^(c)^
AST (IU/L)	41 ± 5	38 ± 4	532 ± 61***	48 ± 4^(c)^
ALT (IU/L)	26 ± 10	28 ± 6	242 ± 43***	36 ± 4^(c)^

HOMA-IR, homeostasis model assessment of insulin resistance; QUICKI, quantitative insulin sensitivity check index; FGIR, fasting glucose-to-insulin ratio. Data are the mean ± S.D. Statistical differences were analyzed by two way ANOVA. *p < 0.05; **p < 0.01; ***p < 0.001 effect of diet; ^(b)^p < 0.01; ^(c)^p < 0.001 effect of surgery.

None of these changes was observed in the HFD-rats that underwent omentectomy (HFD/O-rats). Thus, although their body weight increased significantly more than in control rats, this increase was markedly less than in HFD-rats (Fig. 1a). Moreover, omentectomy prevented or mitigated the development of many MS features, including hepatic steatosis, even though these rats continued on a HFD (Fig. 3d,e). Thus, plasma glucose, triglyceride, and insulin levels, as well as the HOMA-IR index were significantly lower in HFD/O rats than in HFD-rats (Table 1). Moreover, tyrosine phosphorylation of IRS-1 increased, and serine phosphorylation of IRS-1 decreased in HFD/O rats indicating higher insulin sensitivity (Fig. 2). In addition, plasma adiponectin remained at control levels in HFD/O rats (Table 1). In these rats, steatosis was not observed (Fig. 3d,e) and hepatic triglycerides were significantly lower than in HFD-rats (Table 1). Hepatic gene expression of *Tnf, Ccl2, Ifng, Crp, Casp3, Col1a1, Acta2, and Tgfb* was normal in HFD/O-rats (Fig. 4a). Likewise, hepatic TBARS (Fig. 4b), GSH (Fig. 4c), and 3-tyrosine nitrated proteins levels, as well as protein expression of iNOS and CHOP, (Fig. 4d) were also normal in this group of rats.

In HFD/O2-rats, that is, in rats undergoing omentectomy after two months on a HFD, food intake decreased significantly two months after surgery, and weight gain ceased immediately after omentectomy despite continuing on the same diet. Sixty days after surgery, body weight decreased significantly and this decrease persisted throughout the experimental period (Table 2). Likewise, omentectomy was followed by a significant decrease in HOMA-IR and in plasma glucose, triglyceride, and insulin levels (Table 2). Moreover, steatosis grade (Fig. 3f–j), hepatic triglycerides, and inflammation, apoptosis, and fibrogenesis markers also decreased significantly over the months following omentectomy (Table 2). Predictably, SCD2-rats endocrine parameters did not differ from SCD-rats control group. Interestingly, omentectomy in rats on a SCD had no effects on weight gain or on other features of MS (Table 1; Fig. 1a).

**Table 2:** Evolution of some variables following omentectomy in obese rats on a HFD.

Parameter	Rats on a SCD2^(a)^	Rats on a HFD Days after omentectomy						
		0^(b)^	15	30	46	60	75	Sacrifice day
Weight (g)	238 ± 18	416 ± 21	386 ± 18	389 ± 19	387 ± 21	384 ± 16*	370 ± 2 1**	350 ± 17***
Food intake(Kcal/day)	42.32 ± 5.8	64.1 ± 5.7	39.1 ± 14**	56.3 ± 12.6	54.9 ± 10.3	42.8 ± 10.1*	41.8 ± 10.2**	34.2 ± 9.4***
Plasma glucose (mg/dL)	110 ± 3.2	191 ± 6.4	155 ± 4.6***	133 ± 3.5***	120 ± 2.9***	122 ± 3.1***	121 ± 2.6***	129 ± 4.1***
Plasma insulin (ng/mL)	2.2 ± 0.4	5.6 ± 0.7	4.4 ± 0.5*	3.2 ± 0.4***	3.1 ± 0.2***	3.1 ± 0.4***	3.1 ± 0.3***	2.9 ± 0.3***
HOMA	2.2 ± 0.4	10.0 ± 1.2	6.5 ± 0.7***	4.1 ± 0.5***	3.6 ± 0.2***	3.7 ± 0.5***	3.93 ± 0.4***	3.94 ± 0.3***
Plasma Triglycerides (mg/dL)	60.2 ± 8.6	191 ± 14.1	133 ± 9.9***	113 ± 7.8***	65.5 ± 8.3***	48.5 ± 4.7***	40.1 ± 3.8***	43.2 ± 2.6***
Plasma Leptin (ng/mL)	1.4 ± 0.2	21.9 ± 2.4	22.9 ± 3.2	23.7 ± 2.8	9.3 ± 1.7***	9.1 ± 2.4***	8.3 ± 1.9***	5.9 ± 1.6***
TNF-α (pg/mL)	1.8 ± 0.2	18.7 ± 1.2	18.4 ± 1.6	16.1 ± 1.7	9.3 ± 0.6***	9.2 ± 0.3***	5.7 ± 0.1***	5.7 ± 0.1***
IL-6 (pg/mL)	68 ± 0.6	542 ± 10.8	76 ± 2.2***	72 ± 3.4***	70 ± 2.0***	70 ± 2.6***	68 ± 1.5***	69 ± 3.1***
Hepatic Triglycerides (mg/g liver tissue)	42.9 ± 6.3	519 ± 12.4	—	—	—	—	—	65.9 ± 6.1***
Gene expression *Tnf* (-fold)	1	7.2 ± 0.2	—	—	—	—	—	4.2 ± 0.2**
Gene expression *Ifng* (-fold)	1	2.9 ± 0.3	—	—	—	—	—	1.3 ± 0.2***
Gene expression *Ccl2* (-fold)	1	2.9 ± 0.2	—	—	—	—	—	1.6 ± 0.3***
Gene expression *Casp3* (-fold)	1	3.3 ± 0.4	—	—	—	—	—	2.1 ± 0.3***
Gene expression *Col1a1* (-fold)	1	3.3 ± 0.3	—	—	—	—	—	2.2 ± 0.4**
Gene expression *Tgfb* (-fold)	1	2.2 ± 0.3	—	—	—	—	—	1.1 ± 0.2***

^(a)^After two months on a SCD; ^(b)^after two months on a HFD; Results are given as mean ± S.D., *p < 0.05; **p < 0.01; ***p < 0.001 *vs*. day 0 of omentectomy. –fold, change above gene expression in rats on a SCD.

### Omentectomy reduced food intake in HFD-fed rats but not in those fed a SCD

In order to investigate the cause of limited weight gain in rats on a HFD plus omentectomy, we measured food intake and fecal fat. The food intake in HFD-rats receiving high fat diet was 63.0 ± 9.9 kcal/day, which was significantly different from the food consumed by SCD-rats (40.7 ± 5.4 kcal/day). Rats on a HFD that underwent omentectomy (HFD/O-rats) consumed significantly less food than the HFD group of rats without omentectomy (36.9 ± 9.8 kcal/day; p < 0.001) (Fig. 1b; Table 1). When omentectomy was carried out in obese rats on a HFD for two months, food intake decreased 60 days after omentectomy and persisted for the entire length of the observation period (Table 2). In SCD/O-rats, omentectomy did not affect food consumption (40.2 ± 7.9 kcal/day). Fecal triglycerides were not significantly modified in rats on HFD-rats compared to SCD-rats (10.04 ± 2.6 mg/g feces *vs*. 8.47 ± 2.1 mg/g feces) and omentectomy did not modify fecal triglycerides in this rats (9.3 ± 2.4 mg/g feces).

### Effects of omentectomy on the gene expression of *Ghrl, Npy, Agrp, Pmch*, *Lep, Tnf, Il6*, and *Crp* in rats on a HFD

**Figure 5:**
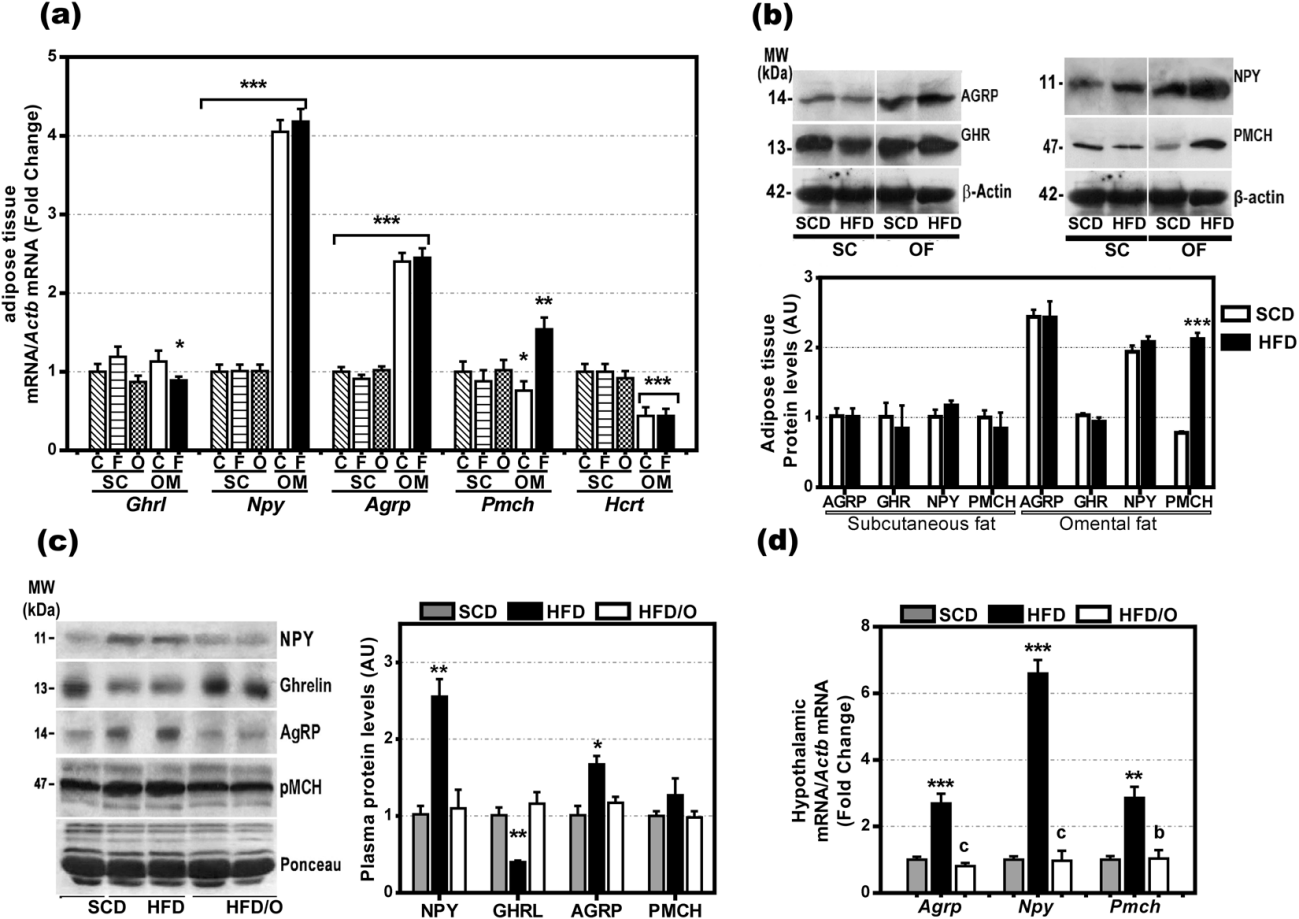
Gene and protein expression of orexigenic factors in omental and subcutaneous fat and hypothalamic gene expression and plasma levels of these factors. (**a**) Gene expression of appetite-stimulatory factors *ghrl, Npy, Agrp, Pmch* and *Hcrt* in the greater omentum (OM) and subcutaneous (SC) fat of rats on a standard chow diet (C) or on a high fat diet (F). *p < 0.05; **p < 0.01; ***p < 0.001 in omental fat as compared with SC fat. (**b**) Protein expression of AGRP, GHR, NPY, PMCH, in the subcutaneous and omental fat of rats fed a SCD or a HFD, respectively. (**c**) Plasma levels of NPY, GHRL, AGRP, and PMCH in SCD-rats, HFD-rats, and HFD/O-rats. Results are given as mean ± S.D. Protein expression in SCD-rats was assumed to be 1. (**d**) Hypothalamic gene expression of *Npy, Agrp*, and *Pmch*. Results are given as mean ± S.D., *p < 0.05; **p < 0.01 ***p < 0.001 *vs*. SCD-rats. ^b^p < 0.01; ^c^p < 0.001 *vs*. HFD-rats.

As omentectomy reduced food intake in HFD/O rats but not in SCD/O-rats, the greater omentum of HFD-rats should contain either orexigenic factors or inhibitors for anorexigenic factors present in neither the SCF nor the greater omentum of SCD-rats. Therefore, we measured gene expression for a number of appetite regulatory factors in the greater omentum and the SCF of SCD-rats and HFD-rats. As Fig. 5a shows, the greater omentum expressed the genes coding for *Ghrl*, *Npy*, *Agrp*, and *Pmch*, four orexigenic hormones. Moreover, the expression of *Npy, Pmch*, and *Agrp* was significantly higher in the greater omentum than in SCF. However, only the gene and protein expression of PMCH was significantly increased in the omental fat but not in the SCF of HFD-rats (Fig. 5a,b).

**Figure 6:**
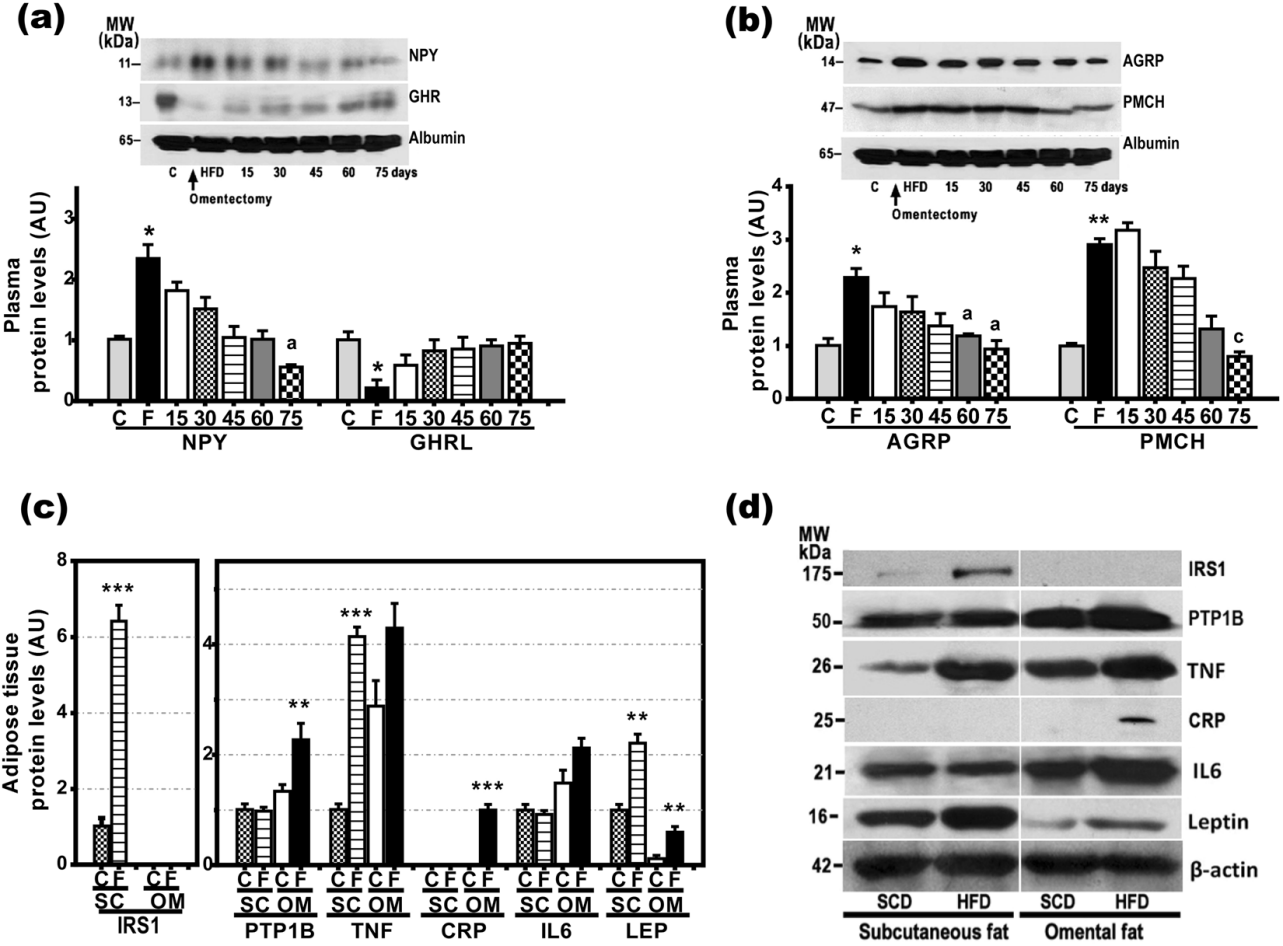
Protein expression of IRS1, leptin (LEP), IL-6, CRP, TNF-α, PTP1B, and insulin (INS) in subcutaneous and omental fat and evolution of plasma levels for orexigenic factors after omentectomy. (**a**,**b**) Evolution of plasma levels of orexigenic factors after omentectomy at the age of 14 weeks in HFD-fed rats (HFD/O2 rats). Bar graphs illustrating protein expression are shown at the bottom of the figures. *p < 0.05; **p < 0.01 *vs*. SCD-rats. ^a^p < 0.05; ^c^p < 0.001 *vs*. HFD-rats. (**c**,**d**) Protein expression of IRS1, PTP1B, TNF-α, CRP, IL6, leptin, and β-actin in the subcutaneous and omental fat of SCD-rats and HFD-rats. Results are given as mean ± S.D., Protein expression in subcutaneous fat of rats on a standard chow diet was assumed to be 1. **p < 0.01 ***p < 0.001 *vs*. SCD-rats.

*Npy*, *Agrp*, and *Ghrl* gene expression in the greater omentum was not higher in HFD-rats than in SCD-rats, while plasma levels of AGRP, PMCH, and NPY were significantly elevated in HFD-rats and correlated to the gene expression in hypothalamus (Fig. 5c,d). GHRL plasma levels were markedly decreased in HFD-rats (Fig. 5c). In HFD-rats that underwent omentectomy at the age of 6 weeks (HFD/O-rats), the plasma levels of all these orexigenic factors remained at control levels (Fig. 5c). Likewise, in HFD/O2-rats, i.e., rats that underwent omentectomy after two months on a HFD and continued with this diet for another 5 months, the plasma levels of all these factors returned to normal within 75 days after omentectomy (Fig. 6a,b).

**Figure 7:**
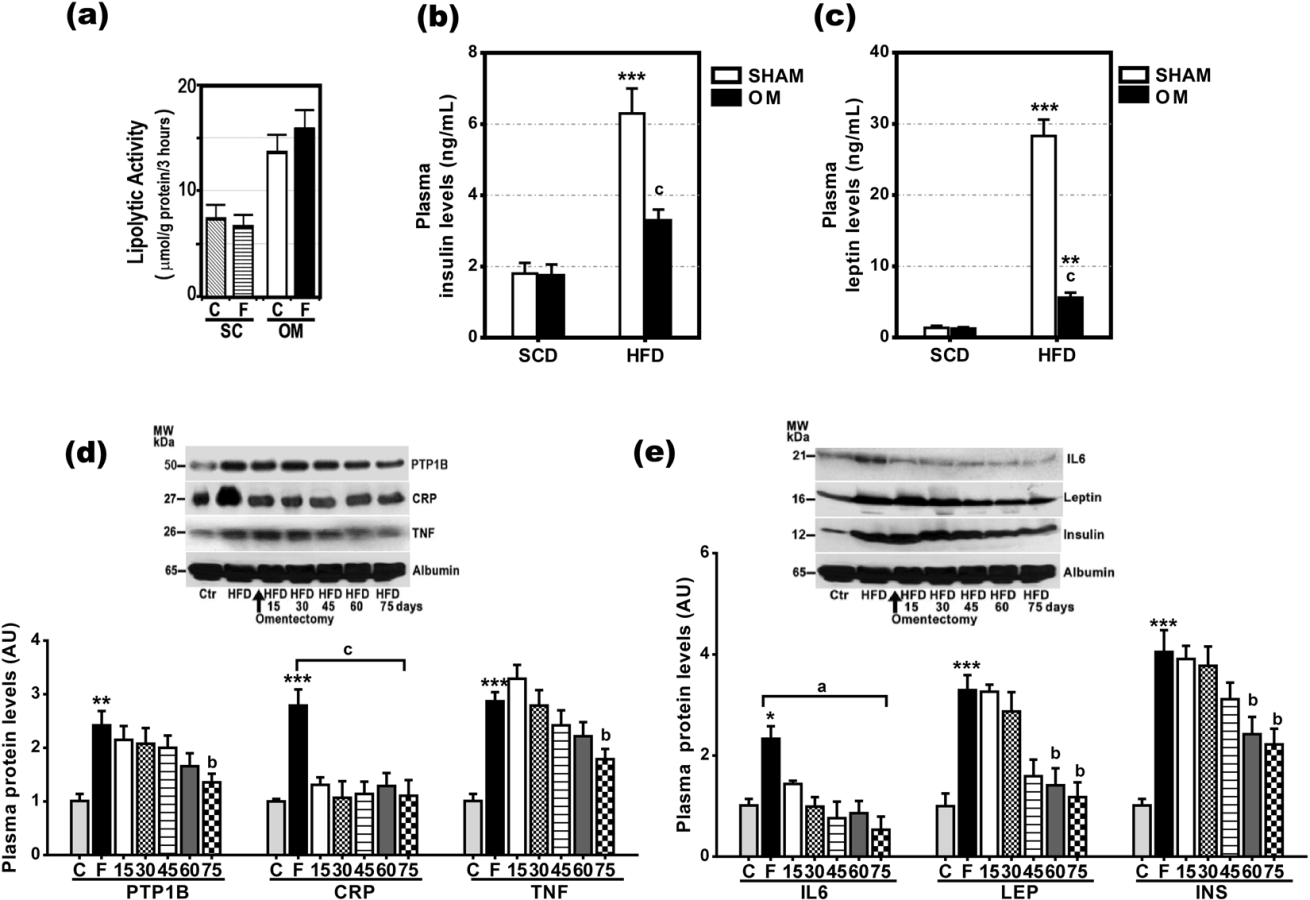
Evolution of plasma PTP1B, CRP, TNF-α, IL-6, leptin, and insulin levels after omentectomy and lipolytic activity of subcutaneous and omental fat. (**a**) Lipolytic activity of subcutaneous (SC) and omental (OM) fat in SCD- (C)- and HFD -fed (F) rats. (**b**,**c**) Plasma insulin (**b**) and leptin (**c**) levels in SCD, SCD/O, HFD and HFD/O-rats. **p < 0.01; ***p < 0.001 effect of diet; (**c**), p < 0.001 effect of surgery. (**d**,**e**) Evolution of plasma PTP1B, CRP, TNF-, IL6, LEP, INS, and albumin levels after omentectomy at the age of 14 weeks in rats on a HFD. Results are given as mean ± S.D. Protein expression in rats on a standard chow diet was assumed to be 1. **p < 0.01 ***p < 0.001 *vs*. SCD-rats. ^a^p < 0.05; ^b^p < 0.01; ^c^p < 0.001 *vs*. HFD-rats.

Because leptin and insulin are hormones that reduce food intake^11^, TNF-α, IL-6, and PTP1B are factors known to cause leptin and insulin resistance, and CRP is a protein that inhibits leptin effects, we also measured protein and gene expression for *lep*, *Ins*, *Tnf, Il6, Ptpn1*, and *Crp* in the omental fat and SCF of SCD-rats and HFD-rats. As shown in Supplementary Figure S1, the gene expression of all these factors, except *Ins*, was significantly elevated in the omental fat of HFD-rats. However, this diet also increased gene expression for *lep, Tnf, and Ptp1n* in SCF. Only the protein expression of IL-6 and CRP was not increased in the SCF of HFD-rats. In fact, the protein expression of CRP was not detected in the SCF and omental fat of SCD-rats (Fig. 6c,d). On the contrary, IRS-1, a key molecule in insulin signaling, was detected in SCF, but not in omental fat (Fig. 6c,d). The absence of IRS1 in the omental fat was associated with increased lipolytic activity in this fat (Fig. 7a). Plasma insulin and leptin levels (Table 1**;** Fig. 7b,c) were markedly increased in obese rats, but significantly lower in the HFD-rats that underwent omentectomy (HFD/O). In HFD/O2-rats, plasma PTP1B, TNF-α, LEP, and INS levels decreased progressively over the next 75 days following omentectomy (Table 2, Fig. 7d,e). In contrast, plasma IL-6 and CRP levels declined rapidly following the resection of the greater omentum (Table 2, Fig. 7d,e).

Despite increased plasma leptin and insulin levels, the activity of both hormones in HFD-rats was likely reduced since these increases were associated with leptin and insulin resistance. Thus, tyrosine phosphorylation of leptin receptors (ObRb) and STAT3 (Supplementary Figure S2), as well as IRS1 (Fig. 2) were markedly decreased in the liver of these obese rats, while serine phosphorylation of IRS1 was elevated (Fig. 2). Likewise, SOCS3, a factor that inhibits cytokine signaling, and PTP1B, a phosphatase involved in inhibiting leptin and insulin signaling by dephosphorylating tyrosine residues^12^, were significantly increased in the liver of high-fat-fed rats (Suppl. Fig. S2). Omentectomy prevented leptin and insulin resistance, as well as the increased liver SOCS-3 and PTP1B levels caused by the HFD in rats (Suppl. Fig. S2).

### CRP inhibits leptin effects in cultured HepG2 cells

**Figure 8:**
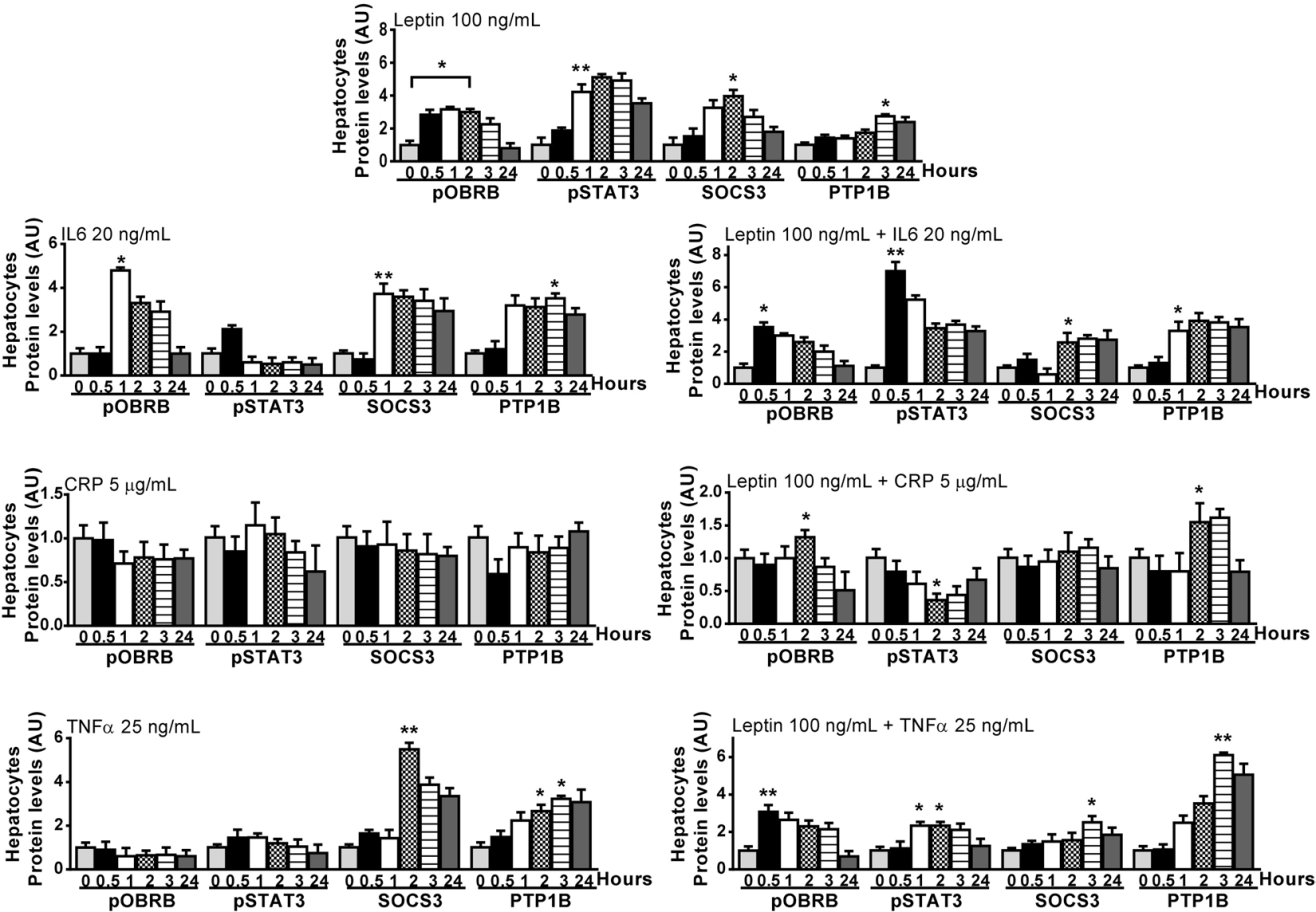
Effects of TNF-α, IL-6, and CRP on leptin signaling. Cultured HepG2 cells were treated with 100 ng/mL leptin in the absence or presence of 20 ng/mL IL-6, 25 ng/mL TNF-α, or 5 µg/mL C-reactive protein for 30 minutes to 24 hours. The effects of these factors were assessed by measuring tyrosine phosphorylation of ObRb and Stat3 and protein expression of SOCS3 and PTP1B, using appropriated antibodies. Protein expression in unstimulated hepatocytes was assumed to be 1. Results are given as mean ± S.D. *p < 0.05; **p < 0.01 *vs*. unstimulated hepatocytes. Representative immunoblots are shown in Supplementary Figure S3.

To determine whether IL-6, TNF-α or CRP, three factors whose gene and protein expression is increased in omental fat, cause leptin resistance, we treated cultured HepG2 cells with 100 ng/mL leptin in the absence or presence of 20 ng/mL IL-6, 25 ng/mL TNF-α or 5 µg/mL CRP for 30 minutes to 24 hours. Then, we measured their effects on tyrosine phosphorylation of OBRB and STAT3, and on SOCS3 and PTP1B protein expression. As Fig. 8 and Supplementary Figure S3 show, the effect of leptin on OBRB and STAT3 phosphorylation was totally blocked in the presence of CRP. Likewise, CRP markedly reduced the effect of leptin on SOCS3 and PTP1B protein expression.

The treatment of HepG2 cells with TNF-α did not prevent leptin from activating the OBRB receptor or STAT3 pathway (Fig. 8, Suppl. Fig. S3). Similarly, this treatment did not reduce the effects of leptin on SOCS3 and PTP1B expression. Finally, the treatment of cells with IL-6 did not affect the binding of leptin to its receptor or the activation of the STAT3 pathway. On the contrary, IL-6 markedly increased phosphorylation of STAT3 at 30 minutes. On the other hand, the effect of leptin on PTP1B increased in the presence of IL-6. Moreover, the increasing effect of leptin on SOCS3 was not prevented in the presence of IL-6 (Fig. 8, Suppl. Fig. 3).

## Discussion

As expected, the consumption of a HFD led to the development of various MS features, including obesity, hyperglycemia, dyslipemia, high levels of plasma insulin, insulin resistance, and NAFLD. Moreover, the gene expression of inflammation, apoptosis, and fibrogenesis markers was increased in the liver of rats fed a HFD. The present study shows that undergoing omentectomy previously the establishment of the diet-induced-obesity markedly reduced the HFD-induced weight gain, and consequently prevented the development of hyperglycemia, hypertriglyceridemia, insulin resistance, and NAFLD (Figs 1, 3 Table 1,).

The limited weight gain caused by omentectomy was due to diminished food intake (Fig. 1b) rather than increased fecal excretion of triglycerides. Interestingly, our study also shows that omentectomy did not change weight gain, food intake or insulin resistance-related parameters in SCD/O-rats (Fig. 1, Table 1). The latter findings are in agreement with the results obtained by Lottatt *et al*., who reported that omentectomy failed to generate measurable changes in body weight in non-obese dogs^13^.

Not many studies have assessed the effects of omentectomy on body weight in animals. While, the role of omentectomy in the staging of obesity is unexplored. In two animal models of obesity, the total removal of the greater omentum resulted in significant improvements in obesity and MS^14^. Similar results have been reported by a number of authors using a variety of animal models^15–18^. It has been recently described that beneficial effects of bariatric surgery on NAFLD are mediated via a decrease in lipogenesis as well as an increase in β-oxidation and autophagy in obese rats^19^. Likewise, patients undergoing omentectomy plus adjustable gastric banding tended to lose more weight than subjects undergoing gastric banding alone. In addition, omentectomy improved oral glucose tolerance and insulin sensitivity, and decreased fasting plasma glucose and insulin levels^7^. However, Fabbrini *et al*. reported that body weight, insulin sensitivity and β-cell function did not change significantly three months after omentectomy in a small cohort of obese subjects with type-2 diabetes mellitus^20^.

More common are studies aimed at determining the effects of omentectomy added to bariatric surgery versus bariatric surgery alone. However, these studies have offered contradictory results. Thus, while several studies have shown that omentectomy added to bariatric surgery resulted in more favorable changes in glucose homeostasis, lipid levels, adipokine profile, weight loss, insulin sensitivity, and β-cell function when compared to bariatric surgery alone^7,21^, other authors failed to confirm these benefits^22^. In a recent multicenter, randomized, controlled trial, the authors found that, two years after surgery, the addition of omentectomy to a Roux-en-Y gastric bypass provided no incremental effect on insulin sensitivity, body mass index, or cardio-metabolic risk factors^23^. These negative results may be explained because the effects of gastric bypass are so marked that the additional effects of omentectomy may go unnotice.

Because omentectomy reduced food intake in rats fed a HFD but not in SCD-rats, we speculated that the greater omentum of HFD-rats might contain an increased amount of orexigenic hormones not present neither in the SCF nor in the omentum of SCD-rats. The measurement of gene and protein expression of several orexigenic hormones revealed the presence of hormones with these effects in the omental fat. Although AGRP, NPY, were markedly higher in omental fat as compared with SCF, however, the exclusion of these hormones by omentectomy is likely not responsible for the reduction in food intake observed exclusively in HFD/O-rats, as the expression of these hormones was at an equally high level in SCD-rats. As indicated, the exclusion of these orexigenic factors by omentectomy did not reduce food intake in these non-obese rats. Only the gene and protein expression of PMCH, another orexigenic factor, was slightly increased exclusively in the omental fat of HFD-rats. Therefore, the removal of omental PMCH production may have contributed to reduce food intake in HFD/O-rats.

The plasma levels of GHRL were decreased in HFD-rats, which should be ascribed to the fact that gastric secretion of this hormone is regulated mainly by gastric content and body weight changes^24^. In contrast, hypothalamic gene expression and plasma levels of NPY and AGRP were increased in HFD rats *vs*. SCD-rats, without correlation with omental gene or protein expression in the greater omentum.

Another option for explaining the anorexigenic effects of omentectomy in HFD/O-rats may be the presence of anorexigenic factor inhibitors in the greater omentum of these rats. Among anorexigenic hormones leptin stands out. This hormone binds to leptin receptors on hypothalamic neurons and reduces NPY, AgRP, and pMCH production^25,26^, consequently decreasing appetite. The effects of leptin on the production of these orexigenic factors by the adipose tissue are not well known; however, the fact that omental fat and SCF contain leptin receptors^27^ suggests that orexigenic factors with this origin may respond to leptin effects. There is evidence suggesting that HFD-rats are leptin resistant. Thus, our study clearly shows that leptin gene and protein expression is highly elevated in the omental fat and SCF of HFD-rats (Fig. 6c, Supp. Fig. S1). Likewise, plasma leptin levels are increased in these rats (Fig. 7c). All these increases may be ascribed to the greater amount of adipose tissue seen in these animals^28^. It is well known that leptin is produced predominantly by this tissue, particularly by SCF^29^, and that it plays a central role in regulating food intake, energy balance, and caloric expenditure^30^. Despite such elevated plasma levels of leptin, NPY, AGRP, and pMCH levels were not decreased, but increased, thus suggesting the presence of leptin resistance. It is well established that obese subjects are leptin and insulin resistant^31^. Likewise, tyrosine phosphorylation of leptin receptors (ObRb), Stat3, and IRS1 was decreased in the liver of these rats in spite of elevated plasma leptin and insulin levels (Fig. 2, Supp. Fig. S2). In contrast, SOCS3 and PTP1B, two factors that inhibits insulin and leptin signaling, were markedly increased in HFD-rats (Supp. Fig. S2). After omentectomy, plasma leptin, insulin (Table 2**;** Fig. 7b,c), NPY, AGRP, and PMCH levels decreased (Fig. 6a,b), which may result a decrease in leptin and insulin resistance. In fact, p_**tyr**_ObRb, p_**tyr**_STAT3, and p_**tyr**_IRS1 were increased, and p_**ser**_IRS1, SOCS3, and PTB1B were decreased in the liver of HFD/O-rats (Fig. 2**;** Supp. Fig. S2). All these changes indicate that HFD-rats are leptin-resistant, and that omentectomy may override this resistance.

Acquired leptin resistance may have several mechanisms, including intracellular inhibitors of leptin signaling, such as SOCS3 and PTP1B^32,33^, or circulating factors, such as CRP, which prevents leptin from binding its receptor^34^. SOCS3 binds to cytokine and hormone receptors, including leptin and insulin receptors, which reduces tyrosine phosphorylation and blocks cytokine signaling^35^. PTP1B, expressed in multiple tissues including the adipose tissue (Supp. Fig. S1), acts as a negative regulator of insulin and leptin signaling by tyrosine dephosphorylating upstream signaling molecules^36^.

Prolonged exposition of cells to leptin effects causes leptin resistance by increasing SOCS3 expression^37^. In addition to leptin, also TNF-α and IL-6 as released by adipocytes and inflammatory cells in the adipose tissue induce SOCS3 expression^38^. Our study shows that the gene and protein expression of IL-6 and TNF-α was increased in the omental fat of HFD-rats (Supp. Figs S1, 6b,d). However, only IL-6 gene expression was increased exclusively in the omental fat of HFD-rats (Supp. Fig. S1). Our study confirms that the treatment of HepG2 cells with IL-6 increases SOCS3 and PTP1B protein expression (Fig. 8, Supp. Fig. S3). Therefore, the removal of the omental source of IL-6 may have contributed to decrease leptin resistance and to reduce food intake. TNF-α, whose gene and protein expression was elevated not only in the omental fat but also in the SCF of HFD-rats, induces leptin and insulin resistance by increasing SOCS3 and PTP1B expression (Fig. 8, Supp. Fig. S3)^39,40^. PTP1B gene expression was significantly elevated in the omental fat of HFD-rats (Fig. 4a). After omentectomy, plasma PTP1B levels decreased gradually almost to control levels (Fig. 7d,e). Likewise, hepatic SOCS3 and PTP1B levels were normal in HFD/O-rats (Supp. Fig. S2), which may be ascribed to an absence of leptin and insulin resistance.

Our study also shows that gene and protein expression of CRP is strikingly increased in omental fat, but not in the SCF of HFD-rats (Supp. Figs S1, 6c,d). In fact, this protein was detected exclusively in the omental fat of HFD-rats. Therefore, omentectomy should eliminate a major source of this protein in HFD-rats. Just like it happens with IL-6, the measurement of plasma CRP levels shows a return to control values soon after omentectomy (Fig. 7d,e). CRP is an acute-phase protein produced mainly in the liver but also in other tissues, including adipose tissue, whose concentration increases with tissue injury or inflammation, and declines when damage subsides^41^. Multivariate analyses have shown that serum CRP levels are associated with abdominal obesity in humans^42^. Our study clearly shows that the treatment of HepG2 cells with CRP inhibits the activation of ObRb and Stat3 by leptin (Fig. 7). Experimental data suggest that CRP inhibits leptin effects by interacting with leptin, which impairs leptin ability to bind its receptor thus causing leptin resistance^34^. Thus, omentectomy would also reduce leptin resistance by removing this leptin inhibitor.

We conclude that omentectomy prevents the development of obesity and metabolic syndrome by reducing food intake in rats fed a HFD. In established obesity, omentectomy eliminates the omental production of inhibitors of the leptin and insulin effects, particularly CRP and IL-6. Other factors including PTP1B and especially TNF-α, slowly return toward normal values, which would contribute to maintain leptin and insulin resistance at the early stages.

Therefore, the exclusion of all these factors by omentectomy may contribute to reduce leptin resistance and consequently food intake. However, the critical role of these inhibitors in causing leptin resistance and low food intake requires further study using animals deficient in these factors. If the effects of omentectomy seen in rats could be confirmed in humans, omentectomy would be a promising procedure to treat or prevent metabolic syndrome, including NAFLD.

## Methods

Five-week-old, female Wistar rats (body weight between 134.0 g and 148.5 g) were purchased from Charles River (Barcelona, Spain). All the experimental protocols involved were approved by the “Ethic and Animal Welfare Commission of University Hospital “12 de Octubre” Madrid, Spain. The animals were kept at constant room temperature (23 °C) under 12-hour light/dark cycles with *ad libitum* access to water and food. After seven days of acclimatization, animals were fed either a standard chow diet (SCD, fat content 11% of energy), or a high-fat diet (HFD) (Harlan Laboratories, Madison, WI) consisting of 21.2% (42% kcal) fat, 17.3% (15.2% kcal) protein, and 35% (42.7% kcal) carbohydrate, for seven months. All procedures were carried out in accordance with the Spanish Guidelines for the Care and Use of Laboratory Animals.

Rats were randomly divided into 6 equal groups of 5 animals each:

1. A control group (SCD-rats), rats fed a SCD that underwent sham surgery at the age of 6 weeks, which consisted of anesthesia, medial ventral laparotomy, manual palpation of the omentum, and analgesic medication.
2. Second group (HFD-rats) were fed a HFD and sham operated as described in the control group.
3. third group (HFD/O-rats) formed by omentectomized rats at the age of 6 weeks and then fed a HFD for the next 7 months.
4. The fourth group (SCD/O-rats) included rats fed a SCD that underwent omentectomy at the age of 6 weeks.
5. The fifth group (SCD2-rats), consisted of rats that underwent sham surgery after 2 months on SCD, and continued for another 5 months on this diet.
6. The sixth group (HFD/O2-rats) included rats that underwent omentectomy after 2 months on HFD, and continued for another 5 months on this diet.

Food consumption and body weight were recorded weekly. One mL venous blood was drawn every two weeks from the jugular vein in HFD/O2-rats and saline was used to maintain blood volume. In all animals, subcutaneous adipose tissue from the inguinal region was analyzed. After overnight fast, animals were anesthetized and sacrificed at 35 weeks of age, and their livers and hypothalamus were rapidly harvested for further analysis. Five to 6 mL venous blood were drawn from the inferior vena cave. A portion of the liver as well as subcutaneous and omental fat was placed in a 4% formaldehyde solution and routinely processed for histological assessment, while the remaining tissue was snap frozen and stored in liquid nitrogen. Sections were stained with hematoxylin-eosin and Masson trichrome. Nitration of mitochondrial proteins by peroxynitrite [3-nitrotyrosine (3-NT)] was assessed by Western blotting as described elsewhere^43^. Small portions of fat, hypothalamus and liver tissue were stored in RNAlater (Sigma, Saint Louis MI) for the measurement of gene expression.

### Omentectomy. Surgical procedure

HFD/O-rats, HFD/O2-rats, and SCD/O-rats underwent omentectomy. Laparotomy was performed through a 2-cm midline incision following isoflurane anesthesia. The greater omentum was removed by meticulous dissection and electrocoagulation of gastroepiploic vessels. The abdominal wall was closed after hemostasis with 4/0 polyglactin 910 (Vicryl, Ethicon, Somerville, NJ) in two layers. The remaining three groups of rats (SCD-rats; HFD-rats; SCD2-rats) underwent sham surgery as described above.

### Calculation of insulin resistance/sensitivity indexes

The following indexes were calculated as estimates of insulin sensitivity: HOMA-IR, QUICKI, and FGIR^44^.

- The homeostasis model assessment of insulin resistance (HOMA-IR) was calculated as the product of fasting plasma glucose and insulin levels divided by 2,430, with insulin given in microunits per milliliter and glucose in milligrams per deciliter. HOMA1 = Glucose (mg/dL) x Insulin (µU/mL)/2,430.
- The quantitative insulin sensitivity check index (QUICKI) was calculated as the inverse log sum of fasting insulin in microunits per milliliter and fasting glucose in milligram per deciliter. QUICKI = 1/[log glucose + log insulin].
- The fasting glucose-to-insulin ratio (FGIR) was calculated as the ratio of glucose divided by insulin levels. FGIR = Glucose/Insulin.

### Cell culture

The HepG2 cell line obtained from American Type Culture Collection (Manassas, VA) was grown at 37 °C in an atmosphere of 5% CO2, 95% air in a cell culture flask using 10 mL of Dulbecco’s Modified Eagle’s Medium containing 10% fetal calf serum, 1% L-glutamine, 1% penicillin, 1% streptomycin, 1% Fungizone. Cells were plated at a density of 5 × 10^6^/80-cm^2^ flask. The effect of **leptin** (Recombinant rat leptin, MBL International Corp., Woburn, MA), **IL-6** (Recombinant rat IL-6, PeproTech EC Ltd., London, UK), **tumor necrosis factor** (**TNF-α**) (Recombinant rat TNF-α, PeproTech EC Ltd., London, UK) and **CRP** (C reactive protein from human plasma, Sigma-Aldrich Chemie GmbH, Schnelldorf, Germany) on phosphorylated ObRb and Signal Transducer and Activator of Transcription-3 (Stat3) and on Suppressor of Cytokine Signaling-3 (SOCS3) and Protein Tyrosine Phosphatase-1B (PTP1B) protein expression was examined between 30 minutes and 24 hours after the addition of these agents to cells cultured in a medium with 1% fetal calf serum.

Lipid peroxidation was determined by measuring thiobarbituric acid reactive substances (TBARS) in cells as described by Ohkawa *et al*.^45^.

Mitochondrial glutathione (GSH) was measured using the Eady *et al*. modification of the Tietze’s assay^46^.

### Western blot

Proteins were separated and transferred to an Immobilon membrane (Millipore, Bedford, MA) as previously described^47^. After electrotransfer, the filters were incubated with the appropriate monoclonal antibody against β-**actin** (Sigma-Aldrich, Alcobendas, Spain) or polyclonal antibody against inducible nitric oxide synthase (**iNOS**), C/EBP homologous protein (**CHOP**), Agouti related protein (**AGRP**), **ghrelin**, neuropeptide Y (**NPY**), melanin concentrating hormone (**PMCH**), **CRP**, **albumin**, **IL-6**, **p**_**tyr**_**1138 Ob/Rb**, **p**_**tyr**_**985 Ob/Rb**, **p**_**tyr**_**Stat3**, **p**_**ser**_**Stat3**, **SOCS3**, **PTP1B** (Santa Cruz Biotechnology, Santa Cruz, CA), **pserIRS**-1 and **ptyrIRS**-1 (Cell Signaling Technology, Danvers, MA), **leptin** (Thermo Fisher Scientific, Waltham, MA), **TNF-α**, and **3-nitrotyrosine** (Abcam plc, Cambridge, UK). Signals were detected using the ECL Western Blotting Detection Reagent (Amersham Ibérica, Madrid, Spain).

### Lipolytic activity

Lipolytic activity was determined in subcutaneous and omental fat using the “Lipolysis Isolation and Quantification Assay Kit” according to the manufacturer’s instructions (Abcam, Cambridge, UK).

### Quantitative real-time polymerase chain reaction

Quantitative real-time polymerase chain reaction was performed following the method described elsewhere^48^. The sequence of primers used in these experiments is shown in Supplementary Table S1. Gene expression of proteins was normalized to that corresponding to β-actin.

### Other determinations

Plasma glucose and aminotransferases were measured using a conventional automatic analyzer. Plasma triglycerides were determined using a triglyceride quantification kit following the manufacturer’s indications (Abcam, Cambridge, UK). Plasma insulin, leptin, adiponectin, TNF-α and IL6 were measured using high-sensitivity ELISA kits. **Leptin**, Rat Leptin Elisa, Merck Millipore Corporation. Billerica, MA; intra- and interassay coefficients of variation were 2.4 and 3.3 respectively. **Insulin**, Rat insulin Elisa Kit (EMD Millipore Corporation, Billerica, MA). **Adiponectin**: Invitrogen, Life technology, Frederick, MD. Intra- and interassay coefficients of variation for measurements of Insulin and adiponectin were 2.9 and 4.0 respectively **TNF-α**, BLK Diagnostics, Badalona, Spain. Intra- and interassay coefficients of variation were 2.4 and 3.6 respectively **IL6**, Rat IL-6 ELISA Kit ThermoScientific, Waltham, MA); intra- and interassay coefficients of variation were 3.5 and 5.1 respectively. Plasma **FFA** levels were determined using the ‘Free Fatty Acids, Half Micro Test’ kit (Roche Diagnostics GmbH, Penzberg, Germany).

### Statistical analysis

All statistical analyses were carried out using SPSS for Windows, Version 21.0 (SPSS Inc. IBM, Amonk, NY,USA). For the presentation of measurable continuous quantitative variables, mean and standard deviation were used. Differences among means were analysed using Student’s t test. Frequencies and percentages were used for categorical data. For comparisons between groups, one-way ANOVA or two-way ANOVA (surgical condition x diet) were performed when appropriate. *p* < 0.05 was accepted as the significance limit.

## Electronic supplementary material

Supplementary Material
