# Supplementary material for: Omentectomy Prevents Metabolic Syndrome By Reducing Appetite and Body Weight In A Diet-Induced Obesity Rat Model
Source: Sci Rep. 2018 Jan 24;8:1540. doi: 10.1038/s41598-018-19973-z (PMC5784083; doi:10.1038/s41598-018-19973-z)
Supplement: Supplementary file 1 — Supplementary Material [file 41598_2018_19973_MOESM1_ESM.pdf]

Title: Omentectomy prevents metabolic syndrome by reducing appetite and body weight in a diet-induced obesity rat model.

Authors: Inmaculada García-Ruiz, Pablo Solís-Muñoz, Daniel Fernández-Moreira, Montserrat Grau, Maria Teresa Muñoz-Yagüe, José A. Solís-Herruzo.

Supplementary Table S1

Primers used in quantitative real-time polymerase chain reaction

Primer name	Direction	Sequence	
**Rat Ghrelin**	**Sense**	5′-GTT CCA GCT TCC TGA GCC CA-3′	NM_021669
	**Antisense**	5′-AAC CTG ATT TCC AGC TCC TCC TC-3′	
**Rat Neuropeptide Y**	**Sense**	5′-GTG GAC TGA CCC TCG CTC TAT-3′	NM_012614
	**Antisense**	5′-TGT CTC AGG GCT GGA TCT CTT-3′	
**Rat Agouti related peptide**	**Sense**	5′-CAA TGT TGC TGA GCT GTG TCC TGC T-3′	NM_033650
	**Antisense**	5′-CTG CTG TCT TCT TCA GAC TTA GAC C-3′	
**Rat Pro-melanin-concentrating hormone**	**Sense**	5′-GAT GAG AGC GGC TTC ATG AAG GAT-3′	NM_012625
	**Antisense**	5′-AAC TCC ATT CAC AGC TGG GAA GAC-3′	
**Rat Tumor necrosis factor-α**	**Sense**	5′-CCT CTT CTC ATT CCT GCT CGT-3′	NM_012675
	**Antisense**	5′-GGC CAT GGA ACT GAT GAG A-3′	
**Rat IL6**	**Sense**	5′-CAC TGC CTT CCC TAC TTC ACA A-3′	NM_012589
	**Antisense**	5′-CAT TCC AAG ATC TCC CTG AGA A-3′	
**Rat Insulin 1**	**Sense**	5′-GTC AAA CAG CAC CTT TGT GGT CCT CA-3′	M_019129
	**Antisense**	5′-TCC AGT GCC AAG GTC TGA AGA TCC-3′	
**Rat Leptin**	**Sense**	5′-GAC CCC AGC GAG GAA AAT GT-3′	NM_013076
	**Antisense**	5′-CCG ACT GCG TGT GTG AAA TGT-3′	
**Rat TGFβ1**	**Sense**	5′-GGA CTA CTA CGC CAA AGA AGT CA-3′	NM_021578
	**Antisense**	5′-CTG GCA CTG CTT CCC GAA TGT-3′	
**Rat IFNγ**	**Sense**	5′-CTC TCT GGC TGT TAC TGC CAA G-3′	NM_138880
	**Antisense**	5′-CCA GTT CCT CCA GAT ATC CAA GAG-3′	
**Rat C reactive protein**	**Sense**	5′-GTC AAA GAA GCC ACT GGA AGC CT-3'	NM_017096
	**Antisense**	5′-CAC CTA CTG CAA TAC TAA ACC CTT GA-3'	
**Rat monocyte chemoattractant protein-1**	**Sense**	5′-CAC AGT TGC TGC CTG TAG CAT-3′	NM_031530
	**Antisense**	5′-GCT TCT TTG GGA CAC CTG CT-3′	
**Rat caspase**	**Sense**	5′-TAC CCT GAA ATG GGC TTG TGT ATA-3′	NM_012922
	**Antisense**	5′-CCA TAA TTT CTT CAC GAG TAA GGT CA-3′	
**Rat collagen α1(I)**	**Sense**	5′-TTC CCT GGA CCT AAG GGT A-3′	NM_053304
	**Antisense**	5′-AAA CCT CTC TCG CCT CTT G-3′	
**Rat α-smooth muscle actin**	**Sense**	5′-GTT TGA GAC CTT CAA TGT CCC-3′	NM_031004
	**Antisense**	5′-CGA TCT CAC GCT CAG CAG TGA-3′	
**Rat protein tyrosine phosphatase-1B**	**Sense**	5′-TGG TGT GGG AGC AGA AGA GCA-3′	NM_012637
	**Antisense**	5′-ACA TCT TCA GAG ATC AGT GTC AGC-3′	
**Rat β-Actin**	**Sense**	5′-GAC GAT ATG GAG AAG ATT TGG CAC-3′	NM_031144
	**Antisense**	5′-CCA GAG GCA TAC AGG GAC AAC ACA GCC-3′	

Supplementary Figure S1

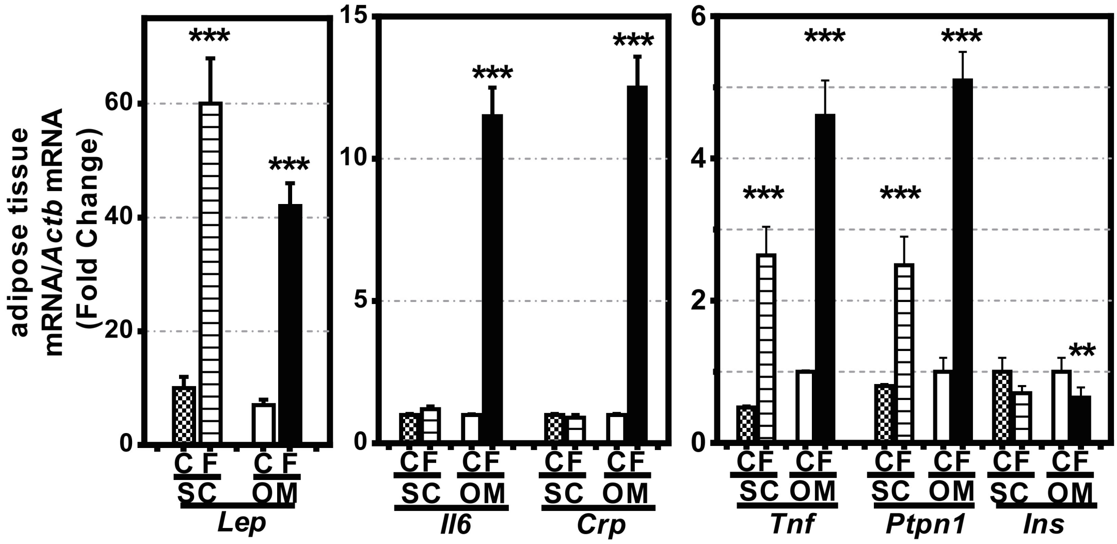

Supplementary Figure S1. Gene expression of *lep, Il6, Crp, Tnf, Ptpn1,* and *Ins* in subcutaneous and omental fat. Gene expression of *Lep, Ins, Il6, Crp*, *Tnf*, and *Ptpn1* in the greater omentum (OM) and subcutaneous (SC) fat of rats under the condition indicated in figure 1A. **, p<0.01; ***, p<0.001 vs. SCD-rats.

Supplementary Figure S2

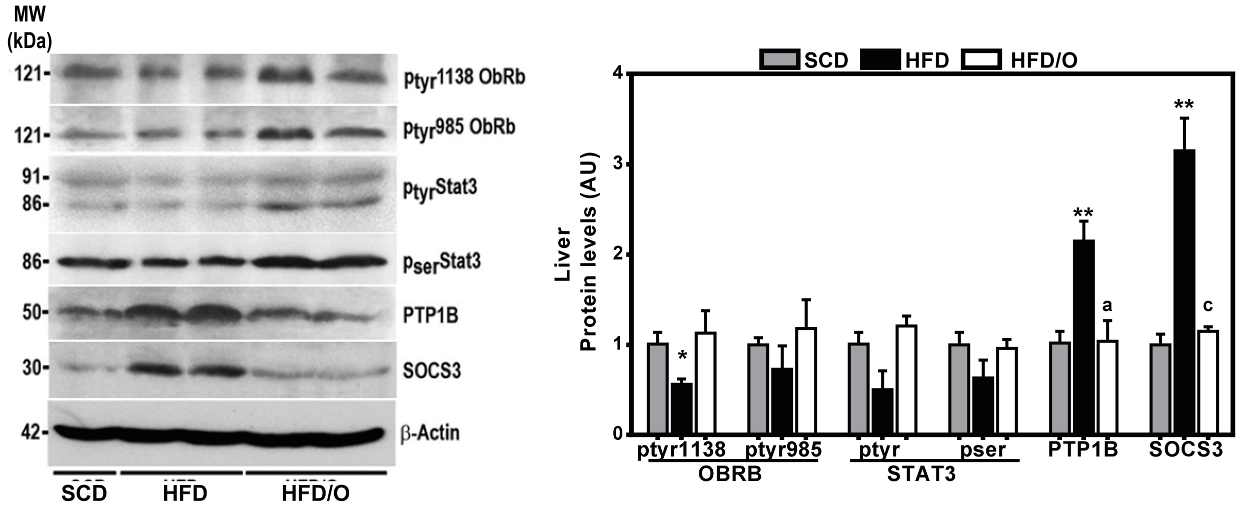

Supplementary Figure S2. Effect of omentectomy hepatic hepatic protein expression of phospho OBRB, phospho STAT3, PTP1B, SOCS3. Effects of a HFD with (HFD/O) or without (HFD omentectomy) at the age of six weeks on hepatic protein expression of phospho-ObRb, phosphor-Stat3, PTP1B, SOCS3, and β-actin. Results are given as mean ±S.D., *, p<0.05; **, p<0.01 vs. SCD-rats; (a), p<0.05; (c), p<0.001 vs. HFD-rats.

Supplementary Figure S3

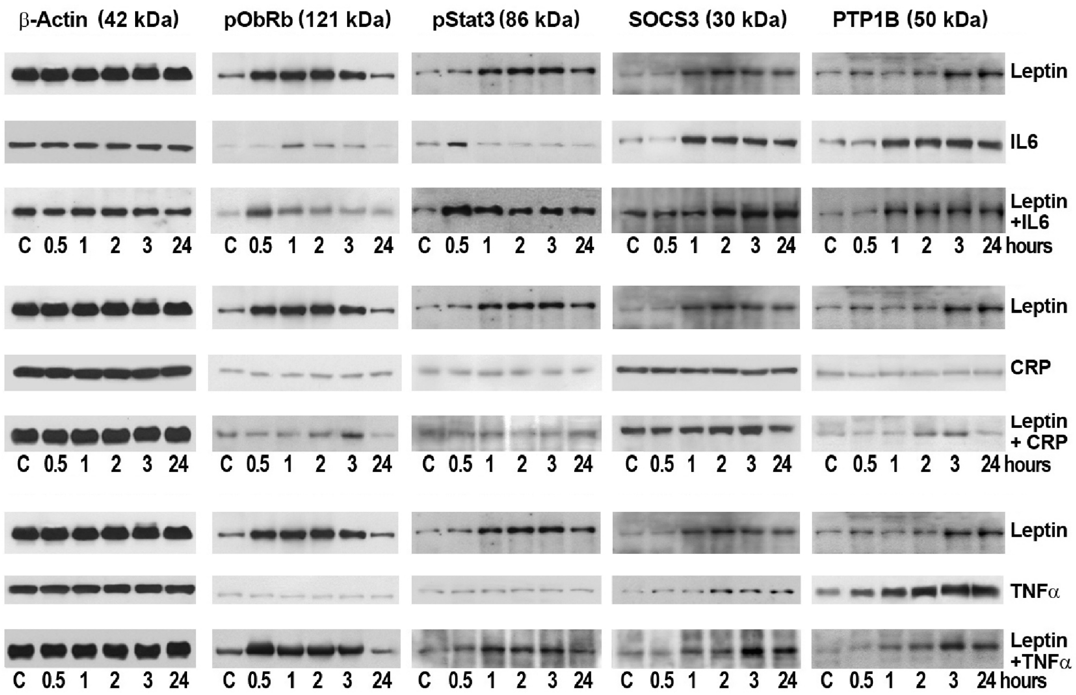

Supplementary Figure S3. Effects of TNFα, IL-6, and CRP on leptin signaling. Cultured HepG2 cells were treated with 100 ng/mL leptin in the absence or presence of 20 ng/mL IL-6, 25 ng/mL TNFα, or 5 μg/mL C-reactive protein for 30 minutes to 24 hours. The effects of these factors were assessed by measuring tyrosine phosphorylation of ObRb and Stat3 and protein expression of SOCS3 and PTP1B, using appropriated antibodies.

Supplementary Figures. Full length blots of Figures 2, 4, 5, 6, 7, S2, S3. Red dotted lines show the cropping locations.

FIGURE 2

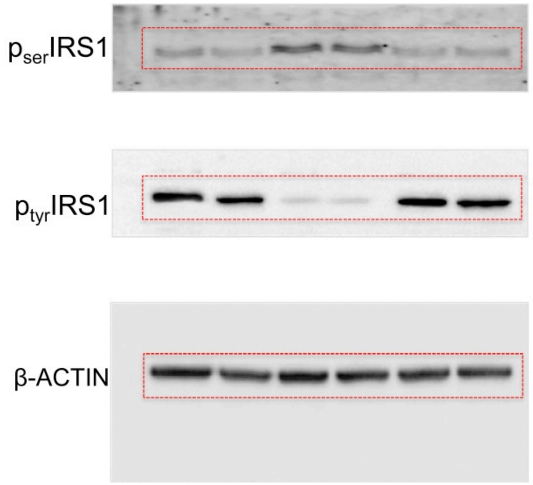

FIGURE 4

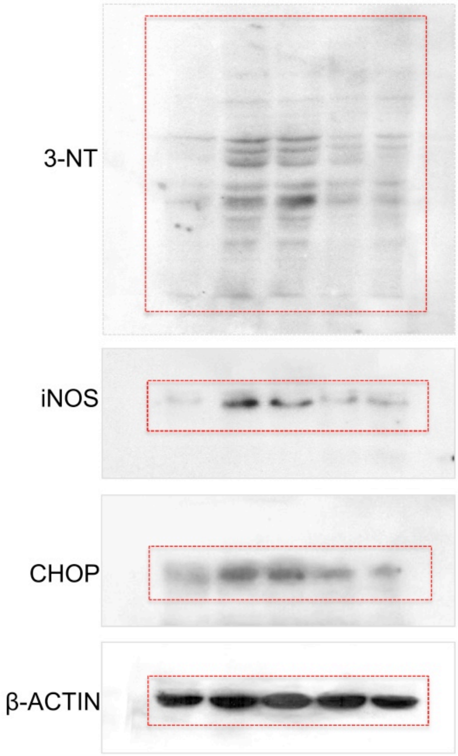

FIGURE 5b

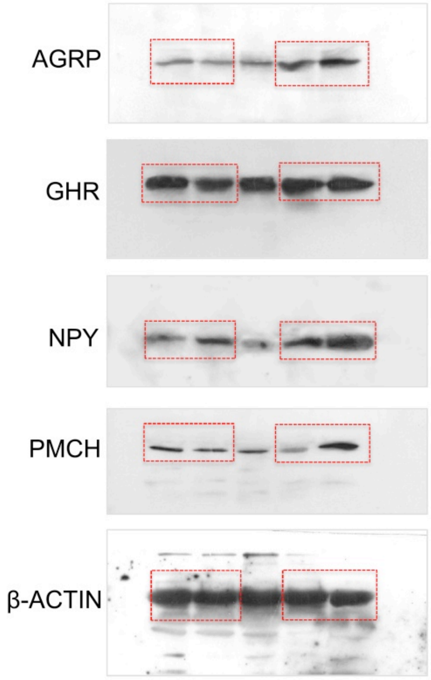

FIGURE 5c

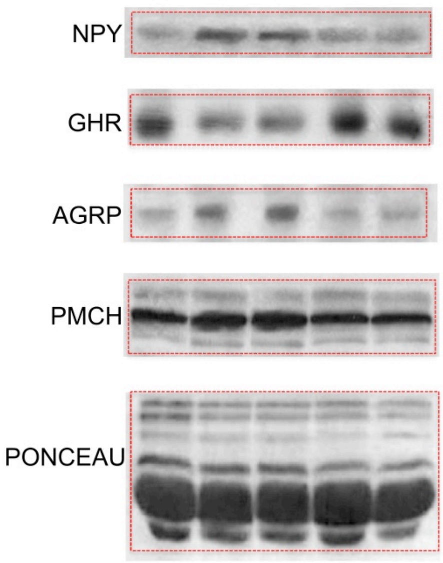

FIGURES 6a, 6b

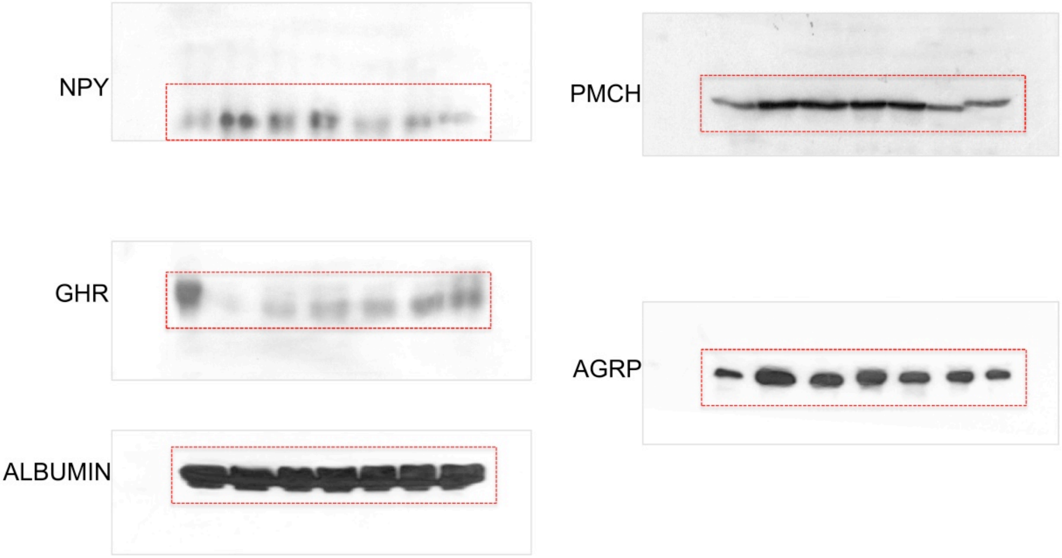

FIGURE 6d

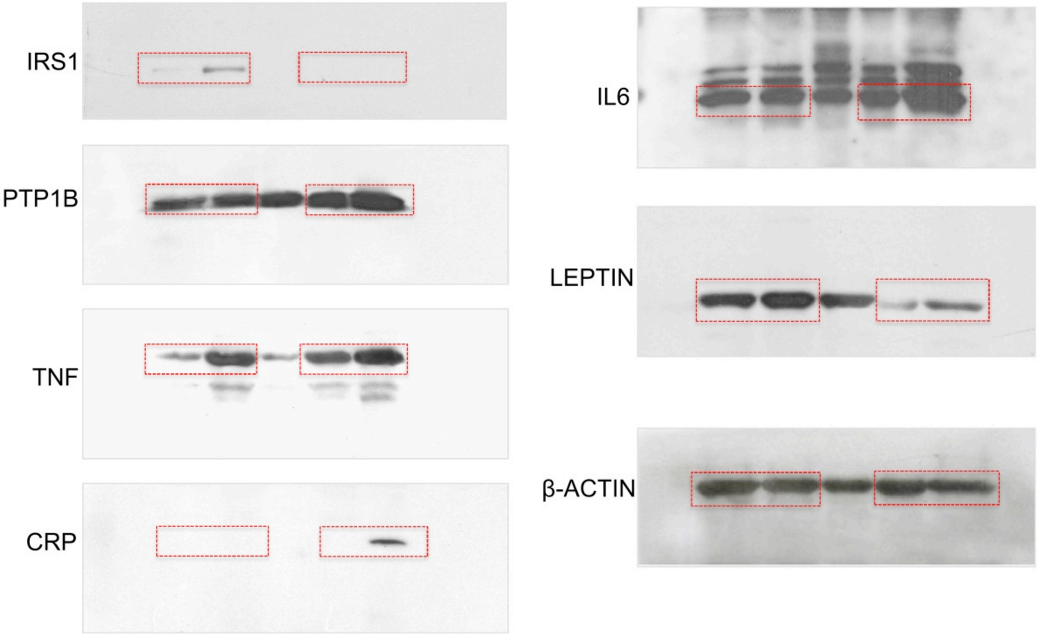

FIGURE 7

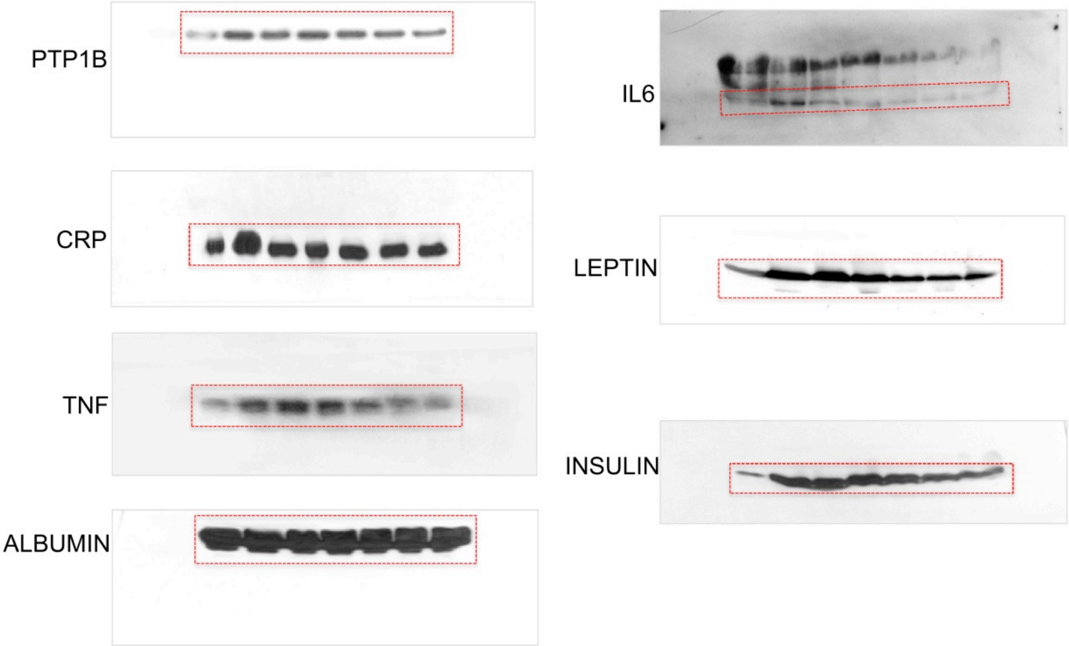

SUPPLEMENTARY FIGURE S2

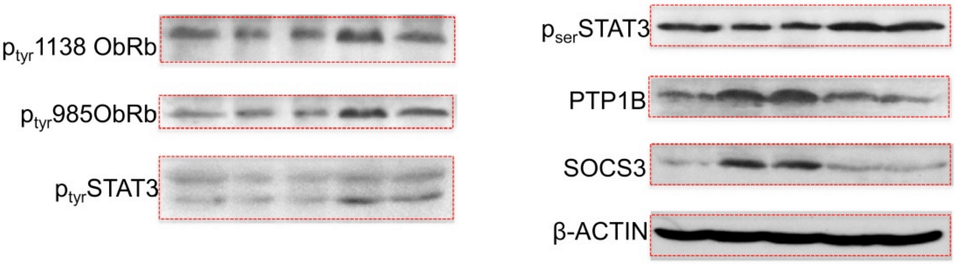

SUPPLEMENTARY FIGURE S3

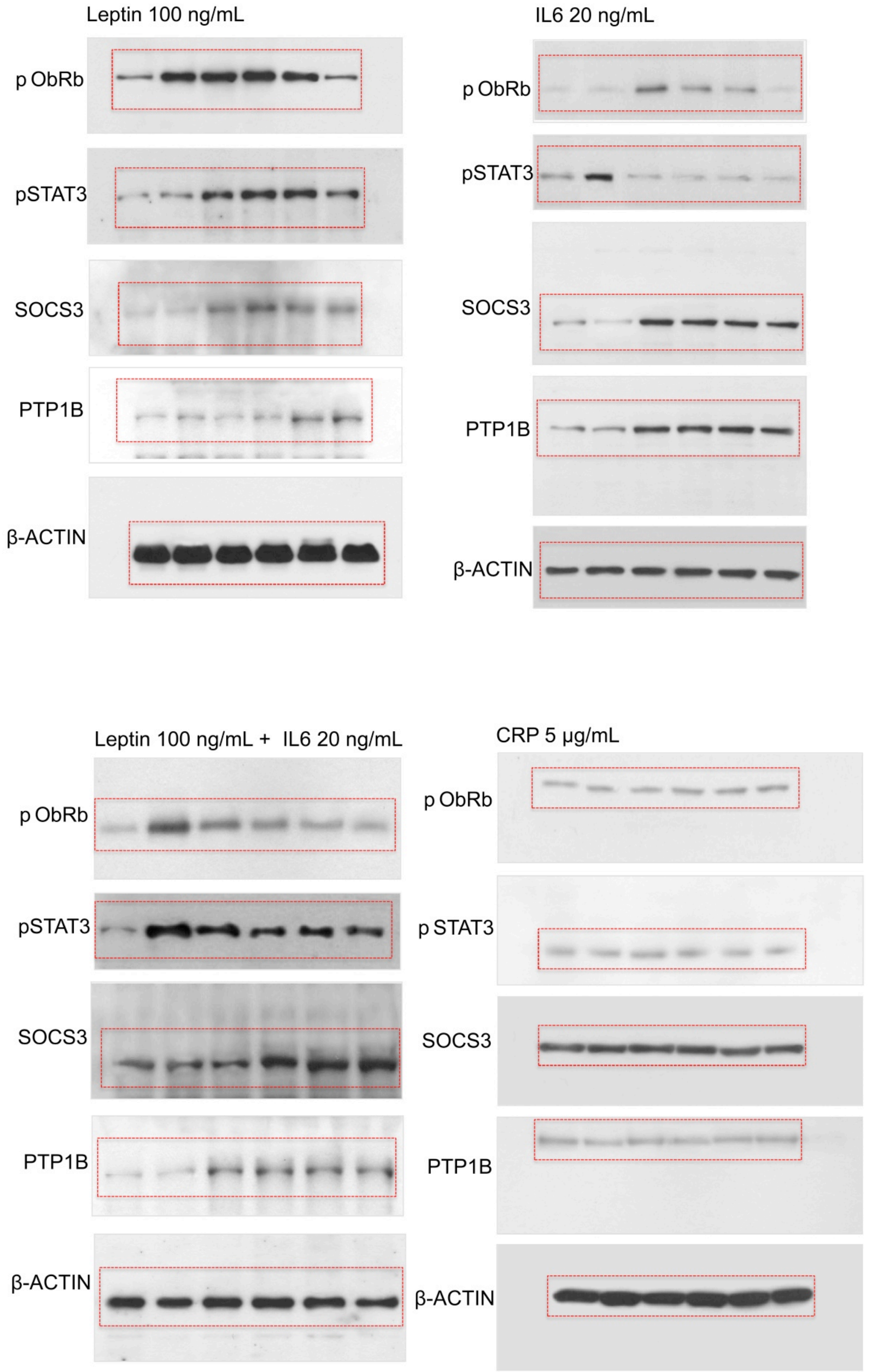

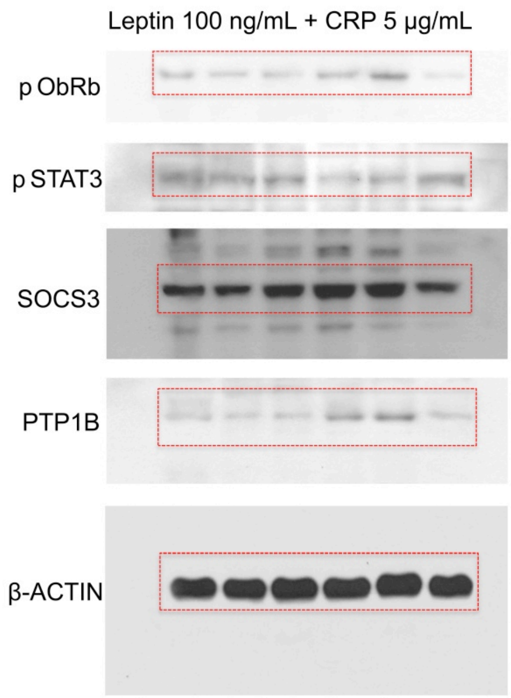
Leptin 100 ng/mL + CRP 5 μg/mL
p ObRb
p STAT3
SOCS3
PTP1B
β-ACTIN

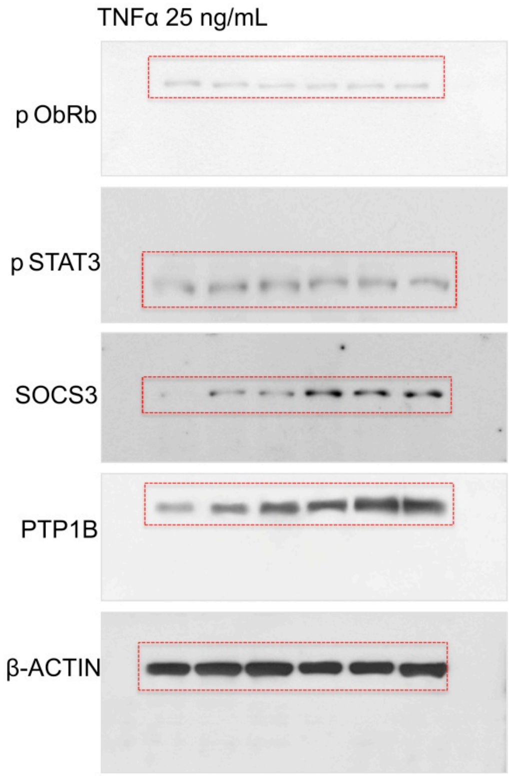
TNFα 25 ng/mL
p ObRb
p STAT3
SOCS3
PTP1B
β-ACTIN

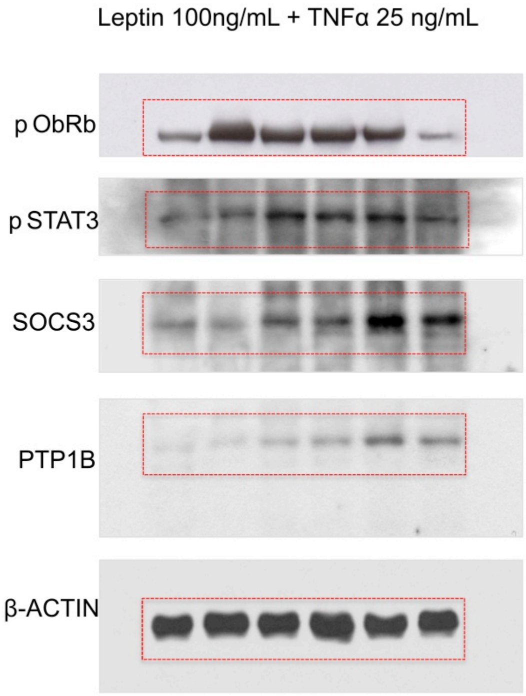
Leptin 100ng/mL + TNFα 25 ng/mL
p ObRb
p STAT3
SOCS3
PTP1B
β-ACTIN
